# Temporal expression of defence and susceptibility genes and tospovirus accumulation in capsicum chlorosis virus-infected capsicum

**DOI:** 10.1007/s00705-022-05401-1

**Published:** 2022-03-04

**Authors:** Fernanda Yuri Borges Naito, Shirani Manel Kumari Widana Gamage, Neena Mitter, Ralf Georg Dietzgen

**Affiliations:** 1 Queensland Alliance for Agriculture and Food Innovation, Centre for Horticultural Science, The University of Queensland, St. Lucia, QLD 4072 Australia; 2 Department of Botany, University of Ruhuna, Matara, Sri Lanka

## Abstract

Yolo Wonder (YW) and Warlock (W), two capsicum cultivars that are susceptible to capsicum chlorosis virus (CaCV), were compared in terms of symptom development, tospovirus accumulation, and host gene expression during the first 12 days post infection (dpi). Temporal expression of selected early CaCV-response genes was used to gain insights into plant-virus interactions and to identify potential targets for CaCV control. Symptoms developed faster in YW during the first seven days of infection, while systemic symptoms were similar in both cultivars at 10 and 12 dpi. CaCV accumulation was higher in YW at 7 dpi despite a lower titre at 3 dpi. At 12 dpi, virus accumulation was similar for both cultivars. Symptom development appears to be correlated to virus accumulation over time for both cultivars. Chalcone synthase (CHS), cytochrome P450 (CYP), and tetraspanin 8-like (TSP8) genes followed a similar expression pattern over time in both cultivars. The thionin gene showed increased expression in CaCV-infected plants at 12 dpi. The WRKY40 gene showed significant differential expression at all time points in YW, but only at 12 dpi in W. The strongest correlation of temporal gene expression and virus titre was seen for CYP, TSP8, thionin, and WRKY40. CHS and CYP may be involved in symptom development, and TSP8 may be involved in virus movement. CHS, CYP, and TSP8 may be good targets for future overexpression or silencing studies to clarify their functions during virus infection and, potentially, for control of CaCV in capsicum.

**Supplementary Information:**

The online version contains supplementary material available at 10.1007/s00705-022-05401-1.

## Introduction

Capsicum chlorosis virus (CaCV) is taxonomically classified as a member of the species *Capsicum chlorosis orthotospovirus*, genus *Orthotospovirus*, family *Tospoviridae*, order *Bunyavirales* [1]. CaCV was first reported in 1999 infecting tomato and capsicum crops in Queensland, Australia [2]. CaCV infects economically important vegetable crops, including tomato in Australia, India, Japan, and Thailand, [2–6], chilli pepper in Australia [2], capsicum in Australia, Greece, and Japan [2, 6, 7], and zucchini and peanut in China [8, 9]. Pineapple as well as ornamental plants and weeds, including *Ageratum conyzoides*, *Emilia sonchifolia*, *Sonchus oleraceus*, and *Tagetes minuta*, are also hosts of CaCV in Australia [10], as are *Rudbeckia* spp. in Iran [11], amaryllis (*Hippeastrum hybridum* Hort), blood lily (*Haemanthus multiflorus* Martyn), and calla lily (*Zantedeschia* spp.) in Taiwan [12, 13], and waxflower (*Hoya calycina* Schlechter) in the United States [14].

Three thrips species have been reported to be CaCV vectors: *Ceratothripoides claratris* [4], *Thrips palmi* [15] , and *Frankliniella schultzei* [16]. Like all orthotospoviruses, CaCV has a negative-sense or ambisense genome composed of three single-stranded (ss) RNA segments, named Small (S), Medium (M), and Large (L). The first Australian CaCV isolate to be completely sequenced was QLD-3432 (KM589495, KM589494, and KM589493). Its S segment is composed of 3944 nt and encodes the nucleocapsid (N) protein and a non-structural RNA silencing suppressor (NSs), its M segment of 4846 nt encodes a glycoprotein precursor (Gn/Gc) and a non-structural movement protein (NSm), and its L segment of 8913 nt encodes an RNA-dependent RNA polymerase (L protein) [17]. This isolate has an unusually large intergenic region (1663 nt) in the S segment [17].

All current commercial cultivars of *Capsicum annuum* are susceptible to CaCV, but resistant breeding lines have been obtained recently. These were generated by transferring the resistance trait from *C*. *chinense* PI290972, followed by several generations of back-crossing [16]. Transcriptome analysis of resistant and susceptible capsicum identified genes that were differentially expressed (DE) in response to CaCV 4 days postinoculation (dpi) [18]. At this early time point, plants of the resistant line showed pronounced necrotic local lesions on the inoculated leaves, indicative of a hypersensitive response, while susceptible capsicum plants did not show any symptoms. Several capsicum genes were found to be up- or downregulated in response to CaCV. In total, there were 2484 DE genes in both resistant and susceptible capsicum infected with CaCV, and of these, 273 were exclusive to the resistant line and 70 were exclusive to the susceptible line [18].

The previous study focused on the genes that were DE in the resistant line to assist in the identification of candidate genes potentially involved in CaCV resistance [18]. Although the functions of the genes were predicted *in silico*, their identity and function in the resistance response to CaCV has not yet been elucidated. Several of the DE genes in susceptible capsicum (cv. Yolo Wonder) are associated with pathogen defence or susceptibility, including chalcone synthase (CHS), cytochrome P450 (CYP), tetraspanin 8-like (TSP8), thionin-like, and WRKY40 [19]. CHS is a key enzyme in the flavonoid biosynthesis pathway; its expression is induced by wounding, pathogens, insect herbivory, and ultraviolet light [20]. Plant P450s produce a diverse set of signalling molecules, growth regulators, plant hormones, and defence-related compounds [21]. TSPs are transmembrane proteins that have crucial and diverse roles in trafficking of vesicles, motility, and differentiation of tissues. They are responsible for maintaining a complex network of signalling molecules and other membrane proteins [22]. Thionins are antimicrobial peptides and have functions in seed germination and packaging of proteins for storage, and they act in signal transduction as secondary messengers [23]. WRKYs are part of a large family of transcription factors that are associated with responses to abiotic and biotic stresses and with various physiological processes [24, 25].

The aim of the present study was to examine the temporal expression of these early plant response genes until the point at which CaCV infection becomes systemic. This will likely identify host genes that may be crucial for virus replication and movement and/or genes that are activated by the plant in defence against CaCV infection. This work provides insights into plant-virus interactions during the first 12 days of CaCV infection and is aimed at identifying potential novel host targets to help control CaCV.

## Materials and methods

### Gene selection and primer design

Five genes associated with pathogen defence or disease susceptibility that were identified previously at 4 dpi as DE in resistant or susceptible capsicum in response to CaCV infection [18] were selected: chalcone synthase (CHS), cytochrome P450 (CYP), tetraspanin 8-like (TSP8), thionin, and transcription factor WRKY40. PCR primers for those genes (Table 1) were designed using Primer3 [26] in Geneious v.9.1.3 [27] based on the contigs assembled in a previous capsicum transcriptome study [18]. The actin gene was chosen as a reference based on results from the previous study [18].

**Table 1:** Sequences of primers used for real-time PCR quantification of gene expression in capsicum RNA extracts

Primer name	Sequence (5' to 3')	Amplicon size (bp)
Actin_F1	GCTGGACGTGACCTAACTG	150
Actin_R1	GCAGTTTCAAGCTCCTGCTC	
CHS_For	GCTTCGACCCTCAGTCAAAC	105
CHS_Rev	GCTCCCTTGTTGTTTTCAGC	
P450_For	TAGTGAAGTCCTCTTGGGACTC	206
P450_Rev	CGTCACAAATTCATCCTCCAACTC	
TSP8_F1	GTGAGCCCAACAAACTGGAC	196
TSP8_R1	CGACGATGAGGAAGATGAGG	
Thionin_F1	AGTGCCCACAACACCTTTTC	152
Thionin_R1	CTGCTGCGAGTGTTTTAGCA	
WRKY40_For	AGTGCAGGGTCAAGGAAGAG	118
WRKY40_Rev	CTGATGAACGTCCAGGCACA	

### Virus inoculation and sample collection

The capsicum cultivars Yolo Wonder and Warlock were grown in a glasshouse at 25°C with 16 h light/8 h dark conditions. Four-week-old carborundum-dusted plants were rub-inoculated with buffer (mock) or CaCV (isolate QLD-3432). Virus inoculum was prepared by grinding a fresh symptomatic CaCV-infected leaf with a mortar and pestle in 10 mM phosphate buffer (pH 7.6) containing 20 mM sodium sulphite. Twelve plants were used for each cultivar: five were mock-inoculated and seven were virus-inoculated. One leaf disc (1 cm diameter) from mock- and CaCV-inoculated plants was collected on days 3, 7, and 12 post-inoculation (dpi) in a 1.5-mL Eppendorf tube, immediately placed into liquid nitrogen, and subsequently stored at -80°C until RNA extraction. The samples at 3 dpi were collected from the inoculated leaf, while the subsequent samples were taken from the youngest systemic leaf.

### RNA extraction, DNase treatment, and cDNA synthesis

Total RNA was extracted from leaf discs using an RNeasy^®^ Plant Mini Kit (QIAGEN), and the RNA concentration was measured using a NanoDrop™ 1000 spectrophotometer (Thermo Fisher Scientific). Extracts were treated using a Turbo DNA-*free*^TM^ Kit (Invitrogen, Thermo Fisher Scientific), and the concentration was measured as above. RNA extracts were diluted to 20 ng/µL for use in real-time PCR as a no-RT control. After DNase treatment, cDNA was synthesized using a SuperScript^TM^ III First-Strand Synthesis System (Invitrogen) and an oligo dT primer. The concentration of cDNA was measured by NanoDrop and adjusted to 20 ng/µL for all samples.

### Quantification of gene expression by real-time PCR

Real-time PCR was done in a Corbett Rotor-Gene 6000 thermal cycler (QIAGEN) using a SensiFast^TM^ SYBR^®^ No-Rox Kit (Bioline). Each reaction contained 1.0 µL (20 ng) of cDNA, RNA, or water, 0.8 µL of each primer (forward and reverse at 10 µM), 7.4 µL of nuclease-free water, and 10 µL of SensiFast SYBR^®^ No-Rox mix (2x) in 20 µL. The PCR cycling conditions were 95ºC for 2 minutes, 40 cycles of 95ºC for 5 seconds, 60ºC for 10 seconds, and 72ºC for 12 or 17 seconds, depending on the primers used, and acquiring data at the last step. RNA samples were used as a no-RT control with actin primers, and water was used as a no-template control for all the primers. Two technical replicates and four and six biological replicates were used for mock and CaCV treatments, respectively. Relative gene expression was calculated as 2^-∆Ct^. The Shapiro-Wilk normality test followed by an unpaired *t*-test was done using GraphPad Prism version 8.0.0.

### Symptom assessment and virus titre

To assess symptom development following CaCV inoculation, photos of the capsicum plants of both cultivars were taken at 3, 5, 7, 10, and 12 dpi, and a symptom severity score was applied, ranging from 0 to 6 (Table 2). Seven CaCV-inoculated biological replicates were used for symptom grading, which were compared to five biological replicates of mock-inoculated plants.

**Table 2:** Symptom grading scale used in this study

Score	Symptom description
0	No visible symptoms; CaCV-inoculated plants show growth and development similar to that of mock-inoculated plants
1	Slight yellowing of the inoculated leaf; development similar to that of mock-inoculated plants
2	Some chlorotic spots in less than 50% of the inoculated leaf area; development similar to that of mock-inoculated plants
3	Chlorotic spots and/or interveinal chlorosis in more than 50% of the inoculated leaf area; no symptoms on systemic leaves
4	Chlorotic spots and/or interveinal chlorosis in more than 50% of the inoculated leaf area; yellowing starting to show on leaves right above the inoculated ones
5	Chlorotic spots, leaf curling and rugose appearance on systemic leaves; reduction in plant development
6	Very strong curling and rugose appearance, appearance of dark green islands in young systemic leaves; plant stunted

CaCV accumulation was quantified by real-time PCR using a SensiFAST™ SYBR^®^ No-ROX One-Step Kit (Bioline) with the primers CaCV IR-F (5'-GCTTGTACATTTAGTTTATCAGGGTTAG-3') and CaCV IR-R (5'- CCAATTTGTTGAATGAGCTAACTTTGG-3'), yielding an amplicon of 157 bp. The same RNA samples that were used to quantify capsicum gene expression were used here at a dilution of 1 ng/µL. For the standard curve, a 795-bp fragment of the S RNA intergenic region of CaCV-QLD3432 was first amplified by PCR using the primers 5'-CACTCATTGTTTGCATGCTGGAA-3' and 5'-ACACTAAAGCTTTGAGAGAAGTTAG-3', cloned into pGEM^®^-T Easy vector (Promega), and sequenced. Then, the plasmid was linearized with *Nde*I and a standard curve was generated using the One-Step kit, the CaCV IR primer set, and a ten-fold serial dilution series ranging from 10 ng to 0.01 pg of DNA. Cycling conditions were 45ºC for 10 minutes, 95ºC for 2 minutes, and 40 cycles of 95ºC for 5 seconds, 61ºC for 10 seconds, and 72ºC for 5 seconds, acquiring data at the last step. The CaCV S RNA copy number was calculated based on the standard curve. A correlation test for virus titre and gene expression was done for each time point using GraphPad Prism, and correlation over time was calculated using the rmcorr package in R and RStudio [28]. Correlation over time between virus titre and symptom score was also calculated using the rmcorr package.

## Results

### CaCV symptom development in susceptible capsicum plants

**Fig. 1:**
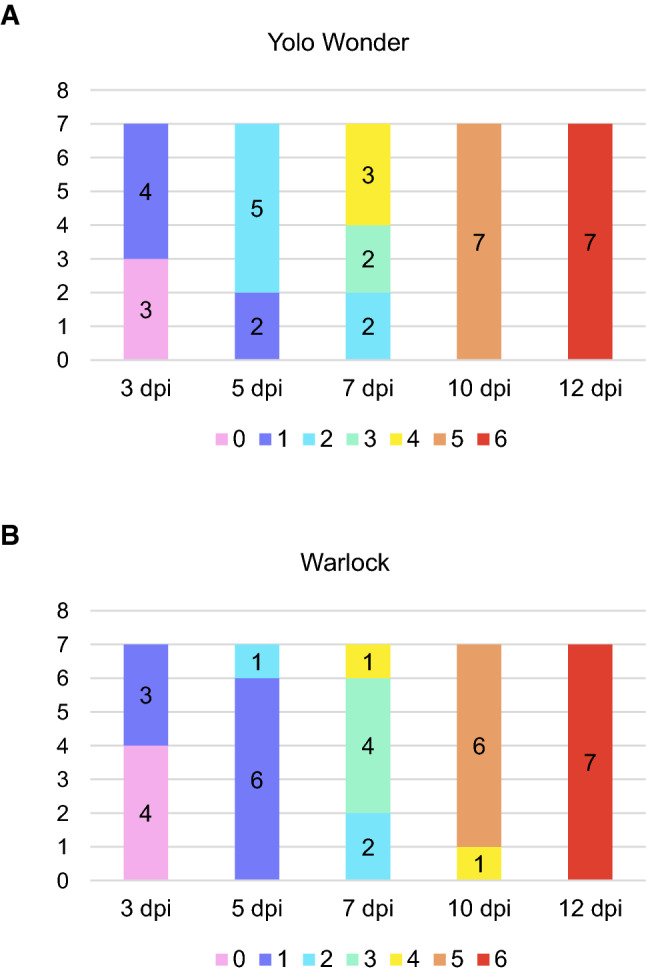
Symptom scores for seven biological replicates at 3, 5, 7, 10, and 12 dpi with CaCV. Different colours indicate different symptom scores. The number of plants showing each symptom is given inside each bar. See Table 2 for the definition of symptom scores. (A) Cultivar Warlock plants inoculated with CaCV. (B) Cultivar Yolo Wonder plants inoculated with CaCV

CaCV symptoms were evaluated at 3, 5, 7, 10, and 12 dpi using a scoring system we developed for capsicum cultivars (Table 2, Fig. 1). Gene expression was analyzed for both cultivars at 3, 7, and 12 dpi. Initial symptoms were slight yellowing on inoculated leaves that progressed to chlorotic spots on less than 50% of the inoculated leaf area without systemic symptoms. The next symptom stage was chlorotic spots on more than 50% of the inoculated leaf area without symptoms on upper leaves. The infection then became systemic, with yellowing on leaves above the inoculated ones. Yellowing on systemic leaves progressed to chlorotic spots, curling, and rugose appearance on younger leaves. The final stage was the development of dark-green islands on systemic leaves and plant stunting (Supplementary Fig. S1).

The first visible symptoms started to appear at 3 dpi, with faint yellowing of a small part of the inoculated leaf area in some plants. At 7 dpi, most CaCV-inoculated plants showed mild symptoms on systemic leaves, and symptoms on the inoculated leaf became more pronounced and occupied a larger leaf area. At 10 dpi, clear systemic symptoms were evident, with some curling and rugosity. At 12 dpi, symptoms were stronger overall, and the plants were stunted. Symptom scores were given to five mock-inoculated and seven CaCV-inoculated plants for each cultivar at 3, 5, 7, 10, and 12 dpi. The number of plants for each score is shown in Fig. 1. None of the mock-inoculated plants showed symptoms, and all of the CaCV-inoculated plants showed symptoms. Individual scores for each biological replicate are listed in Supplementary Table S1.

### CaCV accumulation at different stages of capsicum infection

**Fig. 2:**
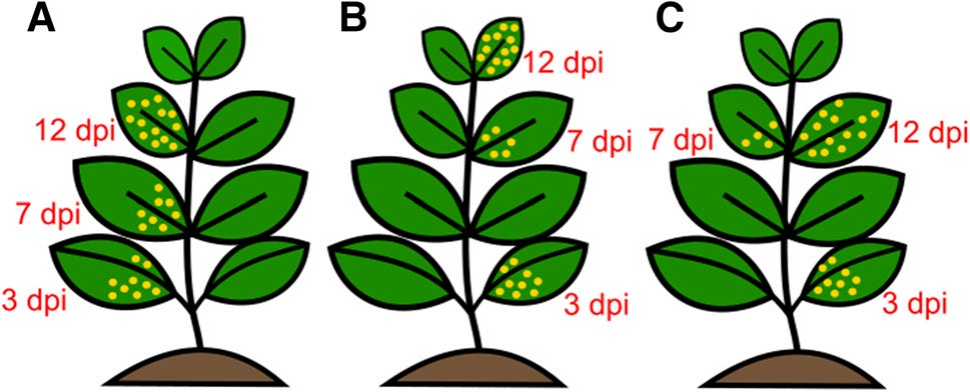
Leaf sample collection diagram for capsicum plants at 3, 7, and 12 dpi. One leaf disc was collected from the inoculated leaf at 3 dpi and the youngest expanded leaf at 7 and 12 dpi. Different leaves were sampled, represented by plants A, B, and C for different biological replicates (Table 3). The number of yellow dots indicates the relative difference in virus titre.

CaCV accumulation was measured by RT-qPCR using a standard curve generated with recombinant plasmid DNA. Titration was done for total RNA from the inoculated leaf at 3 dpi and systemic leaves at 7 and 12 dpi, which were the same leaves selected for the gene expression study (Fig. 2).

**Fig. 3:**
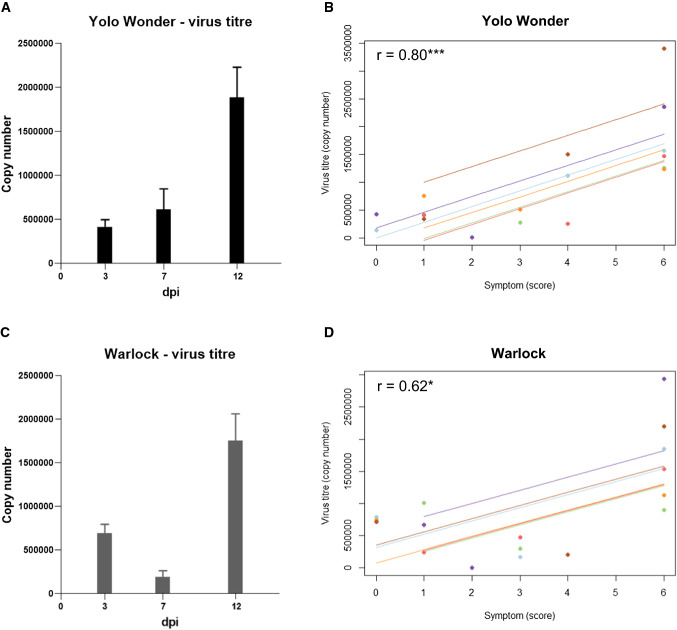
(A and C) Virus titre (copy number/ng tissue) over time in capsicum cv. Yolo Wonder and Warlock. (B and D) correlation between symptom score and virus titre over time for six biological replicates (indicated by different colours) of capsicum cvs. Yolo Wonder and Warlock. Correlation coefficient (r) obtained by repeated measures correlation (rmcorr package), calculated using R and RStudio. Stars represent the statistical significance. ns, not significant (*P* > 0.05); *, *P* ≤ 0.05; **, *P* ≤ 0.01; ***, *P* ≤ 0.001; ****, *P* ≤ 0.0001

The CaCV S RNA intergenic region was targeted to quantify virus accumulation in each test plant to gain an understanding of individual disease progression. At 3 dpi, the number of CaCV copies per ng of tissue in the inoculated leaves of four biological replicates was on average 415,726 for Yolo Wonder and 691,905 for Warlock. At 7 dpi, in the first systemic leaf, the average was 615,105 for Yolo Wonder and 188,989 for Warlock, and at 12 dpi, it was 1,883,435 for Yolo Wonder and 1,756,088 for Warlock. The CaCV copy number showed large differences between individual plants (Table 3). However, overall, the virus titre was higher in Warlock than in Yolo Wonder inoculated leaves at 3 dpi, which was reversed at 7 dpi, when more CaCV had accumulated in systemic leaves of Yolo Wonder. At 12 dpi, both cultivars showed an increase in virus titre compared to 7 dpi, with CaCV accumulating to comparable levels (Fig. 3A and C). Although some differences in virus accumulation could be seen between the two cultivars, these were not statistically significant.

**Table 3:** CaCV copy number per ng of leaf tissue, over time determined by RT-qPCR for six biological replicates each of cvs. Yolo Wonder and Warlock infected with CaCV, and the average for each time point. The sampling type is represented by the letters A, B, and C according to Fig. 2.

Cultivar	dpi	Plant 1	Plant 2	Plant 3	Plant 4	Plant 5	Plant 6	Average	SD (% of average)
Yolo Wonder	3	143,132	406,631	415,016	757,551	424,459	347,564	415,726	47.62
	7	1,116,446	285,286	254,314	515,136	15,904	1,503,544	615,105	93.35
	12	1,563,974	1,264,303	1,475,442	1,236,621	2,359,749	3,400,521	1,883,435	45.04
Sampling type (Fig. 2)		A	B	B	C	A	B		
Warlock	3	782,583	1,004,273	241,667	743,934	669,938	709,037	691,905	36.1
	7	167,151	292,901	470,620	1,831	1,370	200,059	188,989	94.93
	12	1,843,663	894,887	1,530,212	1,125,640	2,939,135	2,202,991	1,756,088	42.59
Sampling type (Fig. 2)		B	A	A	B	A	B

A repeated measures correlation test showed a good correlation between symptom score and virus accumulation over time in Yolo Wonder (r = 0.80), with high statistical significance (*p =* 0.00081). There was also significant positive correlation in Warlock (r = 0.62), with high statistical significance (*p* = 0.023), although it was less strong than in Yolo Wonder (Fig. 3B and D).

### Time course of capsicum gene expression in response to CaCV infection

**Fig. 4:**
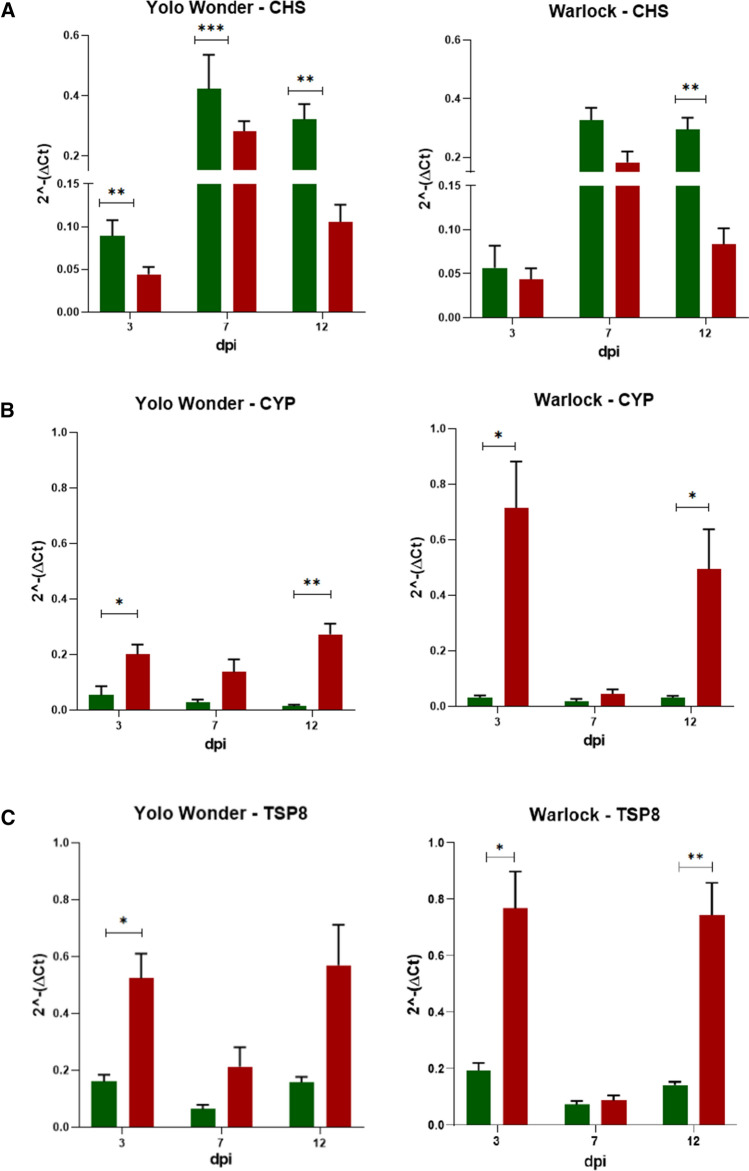

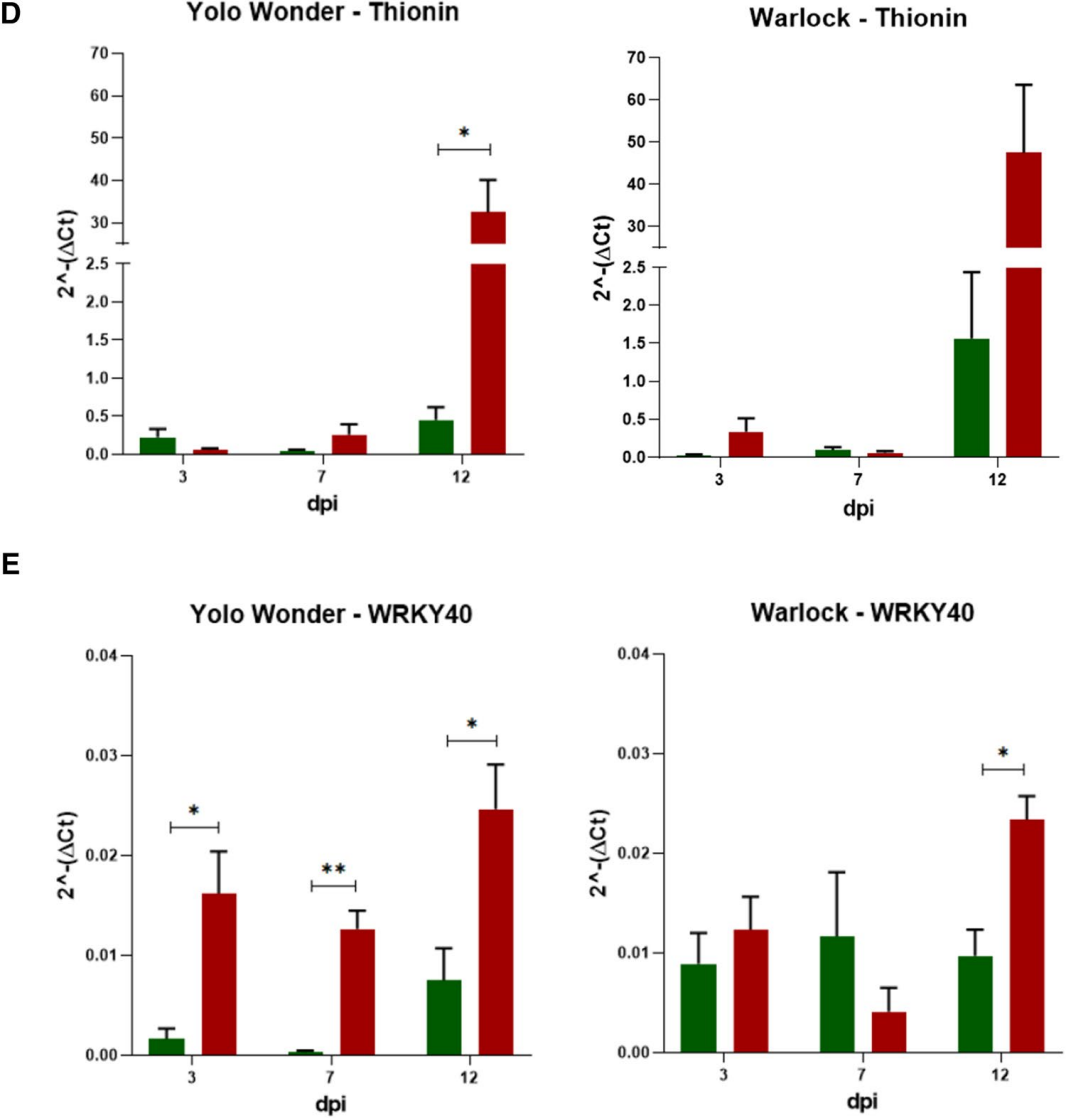
Relative gene expression (2^-ΔCt^) over time in capsicum cv. Yolo Wonder and Warlock. Mock-inoculated plants are shown in green, CaCV-inoculated plants in red. (A) CHS. (B) CYP. (C) TSP8. (D) Thionin. (E) WRKY40. Statistical significance, calculated by the Holm-Sidak method, is shown as stars. ns, not significant (*P* > 0.05), *, *P* ≤ 0.05; **, *P* ≤ 0.01; ***, *P* ≤ 0.001; ****, *P* ≤ 0.0001

Several host genes thought to be involved in plant defence responses were DE at 4 dpi in the susceptible cultivar Yolo Wonder in response to CaCV infection [18, 19]. Of those ‘early response’ genes, five were selected to study their temporal expression in the capsicum cultivars Yolo Wonder and Warlock between 3 and 12 dpi. Relative gene expression is shown in Fig. 4, using different scales according to expression of each gene.

The chalcone synthase (CHS) gene was downregulated in CaCV-infected plants compared to mock- inoculated plants in both cultivars and at all time points (Fig. 4A). CHS gene expression followed the same pattern for mock and CaCV treatments, with increased expression at 7 dpi and reduced expression at 12 dpi. Differential gene expression was statistically significant for Yolo Wonder at all time points when comparing treatments. For Warlock, it was significantly different at 12 dpi only. CHS gene expression and the virus titre in Yolo Wonder were negatively correlated at 3, 7, and 12 dpi individually, although not statistically significant (Table 4).

**Table 4:** Correlation (r) between gene expression and virus titre for five genes and two cultivars at 3, 7, and 12 days post-inoculation (dpi) and over time

Cultivar	dpi	CHS	CYP	TSP8	Thionin	WRKY40
Yolo Wonder	3	-0.6 (ns)	0.75 (ns)	0.84 (*)	0.59 (ns)	-0.16 (ns)
	7	-0.56 (ns)	0.93 (**)	0.69 (ns)	-0.15 (ns)	0.8 (ns)
	12	-0.58 (ns)	-0.12 (ns)	-0.27 (ns)	0.8 (ns)	0.17 (ns)
	r over time	-0.18 (ns)	0.72 (*)	0.32 (ns)	0.87 (***)	0.56 (ns)
Warlock	3	-0.01 (ns)	0.58 (ns)	0.91 (*)	0.00 (ns)	0.29 (ns)
	7	0.08 (ns)	0.6 (ns)	0.92 (**)	0.49 (ns)	-0.23 (ns)
	12	-0.27 (ns)	0.76 (ns)	0.11 (ns)	0.81 (*)	-0.46 (ns)
	r over time	-0.47 (ns)	0.51 (ns)	0.65 (*)	0.89 (****)	0.62 (*)

Stars represent the statistical significance. ns, not significant (*P* > 0.05); *, *P* ≤ 0.05; **, *P* ≤ 0.01; ***, *P* ≤ 0.001; ****, *P* ≤ 0.0001

The cytochrome P450 (CYP) gene was upregulated in CaCV-infected plants compared to mock treatment at all time points. CYP gene expression patterns differed between the cultivars. In Yolo Wonder, mock treatment led to decreased CYP expression over time, while CaCV treatment led to lower gene expression at 7 dpi and higher expression at 12 dpi (Fig. 4B). In Warlock, on the other hand, both treatments showed the lowest expression in systemic leaves at 7 dpi and the highest expression at 3 dpi in CaCV-inoculated leaves. CYP gene expression in Warlock was higher than in Yolo Wonder at 3 and 12 dpi. CYP gene expression in CaCV-infected plants was significantly different from that in mock-treated plants at 3 and 12 dpi for both cultivars. CYP gene expression and the virus titre were positively correlated in Yolo Wonder at 7 dpi and over time. No statistically significant correlation was observed for Warlock (Table 4).

The tetraspanin 8-like (TSP8) gene was also upregulated in CaCV-infected plants, and the temporal expression pattern was similar for both treatments and cultivars, with decreased expression at 7 dpi and increased expression at 12 dpi (Fig. 4C). This gene was significantly upregulated in the presence of CaCV at 3 dpi in Yolo Wonder and at 3 and 12 dpi in Warlock. TSP8 gene expression and the virus titre at 3 dpi were positively correlated in both cultivars, and at 7 dpi in Warlock. A positive correlation over the time frame of the experiment was maintained only for Warlock (Table 4).

Thionin gene expression differed between the cultivars depending on the time point. The common characteristic was that, early in infection in both inoculated and systemic leaves at 3 and 7 dpi, thionin gene expression was low in both mock- and CaCV-inoculated leaves of both cultivars, but there was no significant differential expression between treatments. At 12 dpi, on the other hand, this gene was strongly upregulated in CaCV-infected plants of both cultivars, and the difference between treatments was statistically significant for Yolo Wonder (Fig. 4D). Thionin gene expression and virus titre were positively correlated at 12 dpi for Warlock (Table 4). Over time, there was a strong positive correlation for both cultivars.

The WRKY 40 gene was upregulated following CaCV inoculation at all time points for both cultivars, except for Warlock at 7 dpi, where it was downregulated in CaCV-infected plants (Fig. 4E). The highest gene expression was at 12 dpi for both cultivars. This gene was upregulated at all time points in Yolo Wonder and at 12 dpi in Warlock. It is noteworthy that CaCV accumulation in Warlock at 7 dpi was low, coincident with low WRKY40 gene expression, and both measures were increased at 12 dpi (Fig. 4E). There was a positive correlation between WRKY 40 gene expression and virus titre over time in Warlock (Table 4).

## Discussion

Yolo Wonder and Warlock are two well-known bell pepper capsicum cultivars that are susceptible to CaCV, like all current commercial cultivars. Yolo Wonder (Terranova Seeds) has been widely used for many years, although nowadays it is not commonly grown by farmers in the field. This cultivar is resistant to tobacco mosaic virus (TMV), has been used extensively in research, and was used in a previous transcriptome study by Widana Gamage and colleagues [17, 18] in our research group. Warlock (Seminis^®^, Bayer), on the other hand, is the market leader for this type of capsicum. It is resistant to potato virus Y (PVY), TMV, and bacterial leaf spot. For these reasons, both cultivars were selected to evaluate potential variation in defence gene expression and symptom development in response to CaCV infection.

CaCV symptoms on a variety of plant hosts can include necrotic and/or chlorotic spots or rings on leaves, leaf distortion and mottling, apical necrosis, plant stunting, and yellow stripes (on calla lily) [2, 7, 13, 29]. Although symptoms caused by CaCV infection in capsicum have been reported, symptom development over time and molecular defence responses have not been studied. There is a gap in our understanding of CaCV symptom development and the correlation between virus titre and host gene expression. The aim of this study was to investigate the progress of CaCV infection in capsicum plants to identify host genes involved in the spread of infection and antiviral defence.

Overall, the two capsicum cultivars tested did not show major differences in temporal symptom development. At 3 dpi, both cultivars showed no symptoms or very mild symptoms. At 5 dpi, most Yolo Wonder plants scored 2 on the grade scale, while Warlock plants were at 1. At 7 dpi, Yolo Wonder plants showed stronger symptoms, with most plants scoring 4, while Warlock plants were at 3. At 10 dpi, both cultivars had similar symptom scores with clear systemic symptoms, and at 12 dpi, all Yolo Wonder and Warlock plants were at the same severe stage of symptom development. Based on these observations, Warlock plants under glasshouse conditions appear to develop CaCV symptoms slightly more slowly in the early phase of infection in the inoculated leaves compared to Yolo Wonder. Once systemic virus spread had occurred, both cultivars showed similar rapid symptom development in the young leaves, with rugosity, curling, and plant stunting.

The first time point at which CaCV accumulation was measured was at 3 dpi. At this time, the inoculated leaves of Warlock plants had a 1.6-fold higher virus titre than the inoculated leaves of Yolo Wonder. This may have been due to differential virus uptake from the inoculum. Even though the same inoculum was used at the same time for all of the experimental plants, mechanical rub-inoculation may lead to different amounts of virus or uneven distribution in each inoculated leaf. This is indicated by the variable virus copy numbers between the biological replicates. Another point to be taken into consideration is that some plants developed faster than others, leading to expanded leaves earlier than in others. Since we collected samples from the youngest fully expanded leaves, the physical distance between sampling sites at 3 dpi and 7 dpi varied between plants (Fig. 2). For some biological replicates, the samples at 3, 7, and 12 dpi were collected from leaves immediately above the previous one, and for others, the samples from 7 dpi were collected from the second-youngest leaf. This appears to have affected the virus titres in those samples, which was reflected in the large standard deviation between individual replicate plants. For Yolo Wonder, plants 2, 3, and 4 had lower virus titres compared to plant 1, and samples were collected from the second leaf above the inoculated leaf (3 dpi). In Warlock at 7 dpi, samples from plants 1 and 4 were collected from the second leaf above the inoculated leaf, and these also showed lower virus titres compared to plants 2 and 3, lending support to the above hypothesis. Plants 5 and 6 from both cultivars on the other hand, showed opposite patterns.

Overall, in Warlock compared to Yolo Wonder, CaCV accumulated more slowly during the initial phase of infection before 10 dpi, which may suggest initially slower virus replication and/or movement. In a previous study of PVY infecting potato, it was observed that higher initial virus concentration in the inoculum resulted in higher virus accumulation at 5 and 7 dpi and in faster virus accumulation in systemic leaves [30]. Although CaCV-inoculated Warlock plants initially had higher virus titres at 3 dpi, potentially indicating a higher uptake of virus during mechanical inoculation, virus accumulation at 7 dpi and progression of systemic infection were slower than in Yolo Wonder. This progression of infection is opposite to the results of studies with turnip mosaic virus in *N. benthamiana* and tobacco etch virus in *N. tabacum* [31, 32]. Our observations suggest that cell-to-cell and systemic movement of CaCV is slower in Warlock than in Yolo Wonder early in infection, while virus replication and movement appear to be faster after 7 dpi, leading to similar virus titres in both cultivars at 12 dpi, when symptoms are evident. Virus titre and symptom scores correlated well in both cultivars, with stronger correlation in Yolo Wonder, since the virus copy number started lower and increased over time, while in Warlock, the virus copy number was higher at 3 dpi than at 7 dpi. During the early phase of infection, CaCV accumulated less in Warlock, and plants had fewer symptoms compared to Yolo Wonder. During the systemic infection phase, virus titres increased to similar levels and symptoms became more severe in both cultivars.

CHS gene expression following CaCV infection was downregulated in both cultivars, with lower expression in Warlock than Yolo Wonder in both mock- and CaCV-infected plants when comparing each time point separately. CHS gene expression in Warlock was only significantly different in CaCV-infected plants when comparing to mock-inoculated plants at 12 dpi, while in Yolo Wonder, the expression of this gene in CaCV-infected plants was significantly lower at all time points. There was no significant correlation between virus titre and CHS gene expression in either cultivar (Table 4). Taken together, these results suggest that Warlock has a different initial response to CaCV regarding CHS expression, taking longer to change the expression in response to virus infection, but the expression is not linked to the virus titre – only to virus infection. CHS gene expression is known to be induced through various stimuli, which can originate from the environment, such as light/UV light, or during plant development [20]. CHS is a key enzyme for the synthesis of flavonoids and isoflavonoid-type phytoalexins that act in response to pathogens. There have been several studies in which CHS expression increased in response to fungal infection [33, 34].

Suppression of CHS gene expression in flax resulted in modification of the cell wall arrangement and morphology as well as stem enlargement [35]. The observed decrease in CHS gene expression from 7 dpi to 12 dpi in CaCV-infected plants could be involved in the rugosity appearance and curling of younger systemic leaves at 10 and 12 dpi. Furthermore, the fact that early CHS gene expression in Warlock is not different between treatments may be responsible for the delayed development of local symptoms in this cultivar when compared to Yolo Wonder. A study of CHS gene expression in soybean seeds in response to soybean mosaic virus and cucumber mosaic virus showed that both viruses led to an increase in CHS mRNA accumulation but suppressed CHS expression through posttranscriptional gene silencing [36]. In our previous study of capsicum gene expression in response to CaCV infection, the CHS gene was upregulated in a CaCV-resistant breeding line when compared to the susceptible cultivar Yolo Wonder [18]. CHS gene expression was also measured in the early stages of infection (0 to 60 hours) by rice stripe virus (RSV, genus *Tenuivirus,* family *Phenuiviridae*) in resistant and susceptible rice. In that study, CHS gene expression in susceptible rice showed no significant difference between infected and uninfected plants early in infection. However, at 60 h, CHS gene expression in RSV-infected plants was lower than in uninfected plants [37]. The CHS gene expression pattern in that study was therefore similar to that observed in the present CaCV study. Interestingly, both viruses have negative-sense and ambisense genome segments and belong to the order *Bunyavirales*, which may be related to the similar host responses.

CYP is a diverse enzyme superfamily whose members are present in all plants and most other organisms [38]. CYP has distinct functions in plant defence and development, acting in detoxification and biosynthetic pathways [39, 40]. Like CHS, CYP is important for phytoalexin biosynthesis for plant defence, as well as the biosynthesis of other secondary metabolites and hormones [38]. CYP is also known to regulate the jasmonic acid pathway in response to wounding [41]. Capsicum CYP (*CaCYP*) was upregulated following CaCV infection in this study and differentially expressed at 3 and 12 dpi when compared with mock-inoculated plants. There was a significant positive correlation between virus titre and CYP gene expression over time in Yolo Wonder (Table 4).

*CaCYP1* encodes a cytochrome P450 from *C. annuum L*. Bukanghat that has been shown to be involved in the basal defence response of capsicum to *Xanthomonas axonopodis* [38, 42]. In that study, *CaCYP1* expression was rapidly induced during the first 48 h after infection in a resistant capsicum line. The mock-inoculated line also showed upregulation, but to a lower extent, as shown by northern blot and RT-PCR [42]. In our previous transcriptome study, *CaCYP* was upregulated 8-fold in CaCV-resistant capsicum in response to CaCV infection compared to the susceptible line [18]. The upregulation of *CaCYP* following CaCV infection in the present study supports the conclusion that CYP also plays a role in the defence response to CaCV in susceptible capsicum. In Warlock, the expression of *CaCYP* was higher than in Yolo Wonder at 3 dpi. It is possible that this difference in gene expression is reflected in the slower initial symptom development and virus accumulation in Warlock.

TSPs are transmembrane proteins that participate in various steps of the virus replication cycle [43]. In the present study, TSP gene expression was upregulated at 3 dpi in CaCV-infected Yolo Wonder and at 3 and 12 dpi in Warlock. There was a significant positive correlation between gene expression and virus titre over time in Warlock only, but also at 3 dpi in both cultivars and at 7 dpi in Warlock (Table 4). A study with TSP from *Phaseolus vulgaris* described the localization of TSP on the membrane at the tips of root hairs, as well as in cytoplasmic vesicles and associated with plasmodesmata. Those localizations suggest that TSPs play a role in cell-to-cell communication and trafficking [44]. TSP expression may be responsible for plasmodesmata changes that facilitate cell-to-cell movement of CaCV. A previous study has shown that CaCV NSm, the viral movement protein, is localized in the cell periphery, coinciding with plasmodesmata localization [45]. TSP gene expression was positively correlated with CaCV titre at 3 and 7 dpi for both cultivars. This suggests that TSP is important during the early stages of virus infection. Plasmodesmata proteome and subcellular localization studies have demonstrated the presence of TSPs and their association with membrane microdomains [46, 47]. TSPs form microdomains localized at the plasmodesmata and can interact with other TSPs or with other membrane proteins and play a crucial role in intercellular communication [48]. It has been shown that extracellular vesicles also contain TSPs, and such vesicles participate in the response to microbial infection by transporting signalling molecules and proteins involved in the stress response [49]. TSPs are also present in extracellular vesicles, which act in the defence response against pathogens in animals and plants [22]. A study found that turnip mosaic virus was excreted within extracellular vesicles, and fractions collected from virus-infected samples showed higher TSP levels. Since the extracellular space is connected to the xylem, this could be another way viruses move inside infected plants [50]. To clarify the functions of TSP8 in capsicum during the response to CaCV infection, potential interactions of TSP8 and viral proteins and the effect of silencing TSP8 could be investigated.

Thionins are low-molecular-weight antimicrobial peptides that have toxic effects on various microorganisms [51]. Most thionins can disrupt the cell membrane in a detergent-like interaction or through specific binding to membrane receptors [52–54]. Thionin gene expression in susceptible capsicum in response to CaCV in this study was low in the early stages of infection and increased greatly after establishment of systemic infection at 12 dpi, when this gene was significantly differentially expressed in Yolo Wonder. This increase in expression was also seen in Warlock. There was a strong positive correlation between virus titre and thionin gene expression at 12 dpi in Warlock and over time for both cultivars. In a study with chilli leaf curl virus–infected capsicum, gene expression in tolerant and susceptible lines was analysed at different time points of infection. The thionin gene was upregulated in tolerant plants compared to susceptible plants. Thionin gene expression in the tolerant line increased until 24 dpi and then started to decrease. In the susceptible line, on the other hand, thionin gene expression increased at 36 dpi, becoming higher than in the tolerant line, when the symptoms were strongest [55], similar to what was observed in the present study. To better understand the function of thionin expression in the resistance response to CaCV, it would be useful to overexpress (in susceptible lines) or silence (in the resistant line) the thionin gene to gauge changes in the response of capsicum to CaCV. Although several studies have shown that enhanced thionin expression was involved in resistance to bacterial and fungal pathogens [56–58], it is unknown if a higher level of expression of this gene would improve the defence against virus infection.

WRKYs are transcription factors that bind to DNA, are involved in physiological and developmental processes, and are expressed in response to various biotic and abiotic stresses. WRKY proteins have essential roles in the kinase signalling network and interact with plant resistance proteins for a rapid immune response [59]. In the present study, WRKY40 was upregulated in CaCV-infected capsicum at all time points in Yolo Wonder, while in Warlock this gene was only differentially expressed at 12 dpi when compared with mock-inoculated plants. There was significant positive correlation between gene expression and virus titre over time in Warlock, but not in Yolo Wonder. In fusarium-resistant chickpea, WRKY40 expression was upregulated, while in susceptible chickpea, this gene was not expressed. Furthermore, a defence response was triggered in response to *Pseudomonas syringae* in *Arabidopsis thaliana* overexpressing WRKY40 [60]. A capsicum WRKY transcription factor (*CaWRKYd*) that belongs to the same subgroup as AtWRKY40 was reported to play a crucial role during the hypersensitive response to TMV. In plants with silenced *CaWRKYd*, expression of pathogenesis-related (PR) and hypersensitive-response-related genes was suppressed, leading to higher virus accumulation [61]. According to that study, WRKY40 may be involved in negative regulation of PR genes and therefore may have an effect on pathogen susceptibility. Unlike the results of those studies, the expression of WRKY40 in susceptible capsicum cultivars in the present study showed an opposite effect, as Warlock showed a positive correlation over time between gene expression and virus titre. In our previous study, WRKY40 was not differentially expressed in resistant capsicum when compared to susceptible capsicum infected with CaCV, but it was downregulated in CaCV-infected susceptible capsicum when compared to mock-inoculated plants at 4 dpi [19]. Future gene overexpression/silencing experiments may reveal if WRKY40 has a positive or negative regulatory role in the response of capsicum to CaCV infection.

To study the effects of manipulating these early-response genes to potentially induce CaCV resistance in capsicum, strategies such as overexpression, silencing, or gene editing could be used. CRISPR/Cas9 is an efficient gene editing tool used to modify host genes that could be involved in susceptibility or that are crucial for virus replication [62]. Targeting of the eukaryotic translation initiation factor 4E (*eIF4E*) has been shown to confer resistance to cucumber vein yellowing virus, zucchini yellow mosaic virus, and papaya ringspot mosaic virus-W [63]. Further studies to elucidate the interaction of each DE CaCV gene with possible viral genes will be important to understand their potential roles during infection, especially replication and movement. If an essential domain of a host susceptibility gene can be identified, CRISPR/Cas gene editing technologies may be applied to disrupt the compatibility of this virus-host interaction to generate resistant plants that do not support virus infection [64]. The overexpression of a WRKY transcription factor from cotton resulted in enhanced resistance to TMV and cucumber mosaic virus in tobacco plants [65]. The overexpression of plant PR-related genes can also lead to the development of milder symptoms in virus-infected plants, as shown in a study with RSV [66]. Gene silencing studies have also been used to understand gene functions in response to pathogen infection. For example, silencing of *eIF(iso)4E* in plum resulted in accumulation of siRNAs and conferred resistance to plum pox virus [67].

## Conclusions

In the present study, the progression of visible symptoms in mechanically inoculated, CaCV-susceptible capsicum plants appears to largely correlate with virus accumulation over time. The capsicum early CaCV response genes identified at 4 dpi in a previous report [18] that were further analysed in this study continued to be down- (CHS) or upregulated (CYP and TSP8) during the period of 3-12 dpi in both cultivars, suggesting their importance in the initial and continued host response. Thionin gene expression did not follow this pattern but was initially low and then strongly upregulated at 12 dpi. WRKY 40 was consistently upregulated in Yolo Wonder over time. Increased host gene expression and an elevated virus titre correlated well over time for CYP in Yolo Wonder, TSP8 and WRKY40 in Warlock, and thionin in both cultivars. Future studies may determine if these host proteins interact with viral proteins. CHS appears to be linked to symptom expression, and CYP appears to contribute to the difference between Warlock and Yolo Wonder in initial symptom development. TSP8 may be involved in cell-to-cell or long-distance movement of CaCV through plasmodesmata and extracellular vesicles, respectively. These three genes may represent good target candidates for future studies of host gene editing, overexpression, or silencing to better understand the functions of those genes during virus replication and movement as well as symptom development to help control CaCV.

## Supplementary Information

Below is the link to the electronic supplementary material.

Supplementary file1 (DOCX 4891 KB)

Supplementary file2 (DOCX 18 KB)
