# Supplementary material for: Temporal expression of defence and susceptibility genes and tospovirus accumulation in capsicum chlorosis virus-infected capsicum
Source: Arch Virol. 2022 Mar 4;167(4):1061–74. doi: 10.1007/s00705-022-05401-1 (PMC8964570; doi:10.1007/s00705-022-05401-1)
Supplement: Supplementary file 1 — Supplementary file1 (DOCX 4891 KB) [file 705_2022_5401_MOESM1_ESM.docx]

**Figure S1.** Time-course of symptom development following CaCV inoculation of capsicum cv. Warlock plants, **A** to **I** are leaves from CaCV-inoculated plants and J and K are from mock-inoculated plants. **A** Inoculated leaf with score 0 at 3 dpi; **B** Inoculated leaf with score 1 at 5 dpi; **C** Inoculated leaf with score 2 at 7 dpi; **D** inoculated leaf with score 3 at 7 dpi; **E** systemic leaf with score 4 at 10 dpi; **F** systemic leaf with score 5 at 10 dpi; **G** systemic leaf with score 5 at 10 dpi; **H** systemic leaf with score 6 at 12 dpi; **I** youngest leaf with score 6 at 12 dpi; **J** mock-inoculated leaf at 12 dpi; **K** youngest leaf of mock-inoculated plant at 12 dpi.

C

B

A


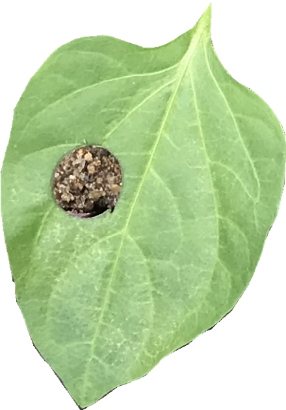

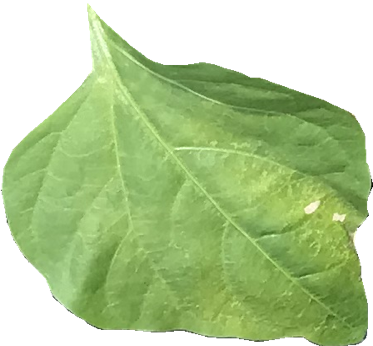

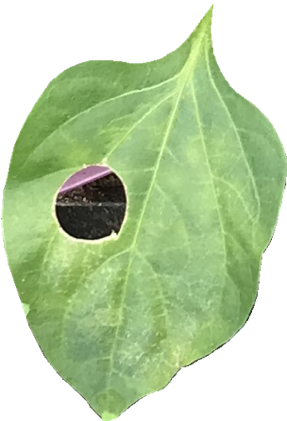

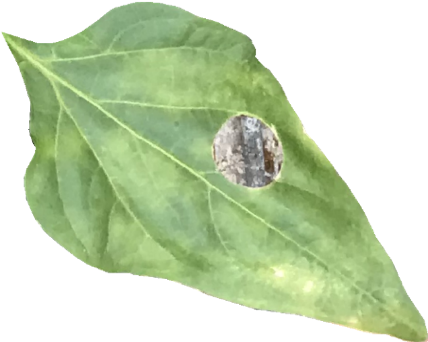

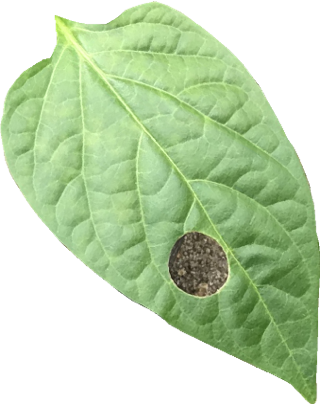

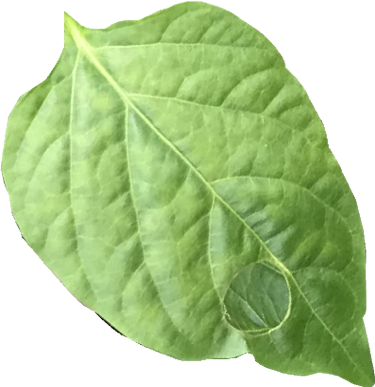

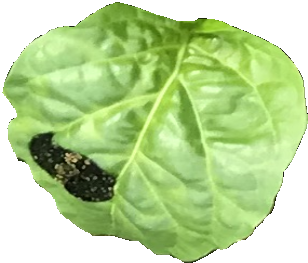

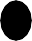

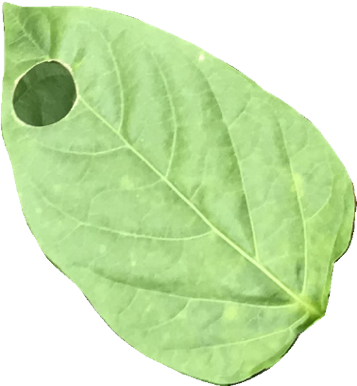

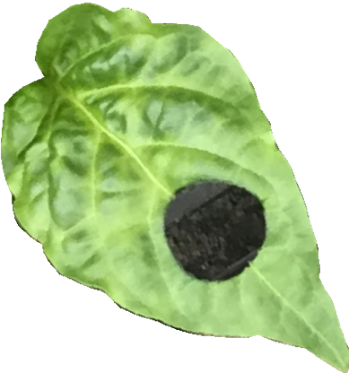

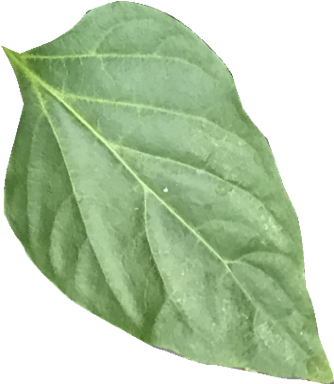

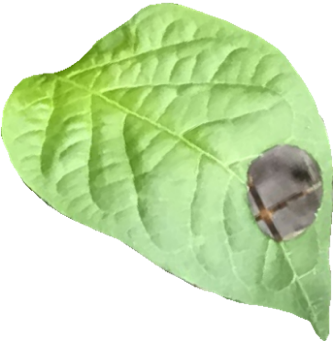


K

J

I

H

G

F

E

D
