# Supplementary material for: Temporal expression of defence and susceptibility genes and tospovirus accumulation in capsicum chlorosis virus-infected capsicum
Source: Arch Virol. 2022 Mar 4;167(4):1061–74. doi: 10.1007/s00705-022-05401-1 (PMC8964570; doi:10.1007/s00705-022-05401-1)
Supplement: Supplementary file 2 — Supplementary file2 (DOCX 18 KB) [file 705_2022_5401_MOESM2_ESM.docx]

**Supplementary Table S1:** Individual symptom scores (Table 2) at different days post inoculation (dpi) with CaCV for seven biological replicates of each cultivar. Symptoms were assessed at 3, 5, 7, 10 and 12 dpi. This data is summarized in Figure 1.

| **Biological replicate** | | | | | | | | |
| --- | --- | --- | --- | --- | --- | --- | --- | --- |
|  | **dpi** | **1** | **2** | **3** | **4** | **5** | **6** | **7** |
| **Yolo Wonder** | **3** | 0 | 1 | 1 | 0 | 1 | 0 | 1 |
|  | **5** | 2 | 2 | 2 | 1 | 2 | 1 | 2 |
|  | **7** | 4 | 3 | 4 | 2 | 3 | 2 | 4 |
|  | **10** | 5 | 5 | 5 | 5 | 5 | 5 | 5 |
|  | **12** | 6 | 6 | 6 | 6 | 6 | 6 | 6 |
| **Warlock** | **3** | 0 | 1 | 1 | 0 | 0 | 1 | 0 |
|  | **5** | 1 | 2 | 1 | 1 | 1 | 1 | 1 |
|  | **7** | 3 | 3 | 3 | 3 | 2 | 2 | 4 |
|  | **10** | 5 | 5 | 5 | 5 | 4 | 5 | 5 |
|  | **12** | 6 | 6 | 6 | 6 | 6 | 6 | 6 |
